# Microwave-sonication synergistic extraction of dairy waste proteins: A review of green approach for dairy waste proteins valorization

**DOI:** 10.1016/j.ultsonch.2024.107111

**Published:** 2024-10-16

**Authors:** Muhammad Waseem, Muhammad Rizwan Javed, Khubaib Ali, Muhammad Saleem, Muhammad Faisal Manzoor, Muhammad Farhan, Robert Mugabi, Aanchal Sharma, Gulzar Ahmad Nayik

**Affiliations:** a Department of Food Science and Technology, Faculty of Agriculture and Environment, Islamia University of Bahawalpur, 63100, Pakistan; b State Key Laboratory of Food Science and Resources, National Engineering Research Center for Functional Food, National Engineering Research Center of Cereal Fermentation and Food Biomanufacturing, Collaborative Innovation Center of Food Safety and Quality Control in Jiangsu Province, School of Food Science and Technology, Jiangnan University, 1800 Lihu Road, Wuxi, Jiangsu 214122, China; c Guangdong Provincial Key Laboratory of Intelligent Food Manufacturing, School of Food Science and Engineering, Foshan University, Foshan, China; d Faculty of Sciences and Technology, ILMA University, Karachi, Pakistan; e Department of Food Technology and Nutrition, Makerere University, Kampala, Uganda; f University Centre for Research and Development, Chandigarh University, Gharuan, Mohali 140413, Punjab, India; g Marwadi University Research Centre, Department of Microbiology, Marwadi University, Rajkot, Gujarat 360003, India

**Keywords:** Microwave, Green extraction, Dairy waste, By products, Whey, Value addition

## Abstract

Ultrasonic and microwave extraction process has great prospects to convert food and agricultural waste from food industries to value-added goods. Also, this review extensively elaborates the utilization of ultrasonication and microwave extraction (US-MW) process for valorization of dairy waste extracted proteins into novel foods. Both of these extraction and processing techniques are considered as green technologies when compared with the other conventional or chemical extraction and processing techniques. Further, this review also explains the impact of US-MW alone and in combination on the dairy waste proteins extraction, nutritional and techno-functional attributes of these dairy-waste proteins. The review also highlights the economic and cost-effective benefits of US-MW processes for extracting the proteins from dairy waste, indicating their feasibility and sustainability. The review also elucidated the synergistic utilization of US-MW extraction as a viable processing technique in extraction or production of bioactive compounds like dairy proteins. In conclusion, this review elucidates the US-MW, both individually and in synergy as a viable source of dairy waste proteins extraction and their application in functional foods. Moreover, in accordance to the latest developments and future prospects at pilot and commercial level to assess the practicability of synergistic use of US-MW extraction in bioenergy production from food wastes other than dairy waste for extraction and production of biodiesel, hydrogen, green methane, and ethanol.

## 1 Introduction

Dairy wastes generated inside the dairy industries contribute to significant environmental issues such as land degradation, water pollution, loss of bio-diversity, deforestation and emissions of green-house gases [1]. The wastes are comprised of high levels of organic and biogenic compounds [2], [3], [4] have estimated that one liter of milk produces up to 10 dm^3^ of effluent. Kolev Slavov [5] stated that whey obtained from cheese, upholds 0.05 % fat, 0.8 % protein and considerable magnitudes of lactose, leading to relatively high biological oxygen demands (BOD) of 30–50 g/L. Buttermilk, a product of churning of butter, have comparatively higher concentrations of fat, proteins and BOD values equivalent to whey. The composition includes approx. 0.5 and 3 % of fat and proteins, respectively. It also contains an appreciable amount of essential nutrients which can be recuperated on further processing [6]. In cheese making process, fines and curd particles are produced and these serve as the major compositional fractions in dairy wastes. These particles are mainly made up of proteins, and fats; this has resulted into high chemical oxygen demands (COD) in the wastewater. It is generated from washing activities in the dairy establishments and it comprises of milk residues, cleaning compounds and disinfectants. The load of organic matter generated from cleaning wastewater is relatively high i.e., BOD > 20 g/L [7]. Apart from proteins, fats and carbohydrates dairy waste contains other nutrient such as nitrogen and phosphorous. If not properly regulated these nutrients can lead to eutrophication [8].

Ryder et al. [9] also revealed, the casein enriched dairy wastewater is received in abundant concentrations which can be valorized in formation of transparent, flexible and tasteless films. Also, milk proteins possess great surface activities and can be used in food industries for the production of ice creams, fermented and non-fermented dairy beverages, formulated meat products, margarine, desserts and puffed snacks. To extract proteins from the dairy wastes is beneficial in improving the cause of sustainability and better economy. Use of dairy wastes into valuable food products, can mitigate the environmental burden produced due to the effects of dairy processing. Resultantly, reduce the utilization of hazardous solvents and energy consuming practices [10]. They enhance sustainability of the dairy industry because they help in minimizing waste and the management of resources [11].

The dairy industry produces substantial amounts of wastes which has high levels of biologically active compounds, including proteins, lipids, fats, and carbohydrates, phenolics, flavonoids and other health-beneficial compounds. However, even under treatment, the quality of dairy waste is still quite high and beyond legal levels thus posing non-compromising impacts on the environment. Hence it is pertinent to render and execute proper and efficient treatment to the dairy waste before it is released to the environment [12]. These macronutrients are now being sought for recovery from foods because of their contributions to the functional attribute and quality of functional food products as elucidated by [13]. Milk proteins for instance are complete protein since they contain all the essential amino acids in the right proportions that man requires in his diet. They are often referred to as protein gold standings because of their relatively high branched-chain amino acids such as valine, isoleucine and leucine, which are more in this products than in other food stuffs [11]. Several extraction techniques are used to extract. The desirable product from food waste since they are a blend of protein, sugar, lipid, vitamin, aromatic compound, phenolics and several antioxidants [14]. New approaches have emerged on protein extraction where the use of microwave-assisted extraction (MAE) [15], [16]; ultrasonication, and pulse electric fields have been developed [17]. Such technologies hold promising prospects for conversion of dairy waste proteins into human food, animal feed and bioplastics [12]. These have been used in the improvement of bakery products, meat and fish, ice cream, yogurt, beverages and fermented drinks and also in the application of fat in low fat type.

This has an impact in the food industry specifically in the aspect of extraction technologies in that there is increase energy efficiency, decrease pollution and increase quality of extracts. Ultrasound assisted extraction (UAE), microwave assisted extraction (MAE), subcritical and supercritical fluid extraction (SFE) and enzyme assisted extraction (EAE) are modern extraction techniques which offer numerous advantages over the conventional solvent extraction technique. These benefits are; less energy and solvent utilization, higher extraction efficiency, more quality extracts obtained, less time used in extraction as well as more easily manageable operations. Observations have revealed that the integrated microwave and ultrasound assisted extraction has benefits in terms of yield and energy savings. The extraction with microwaves of polar components is quite efficient, even more so that the yield with the help of an ultrasound increases considerably. These techniques can be applied in a single reactor or in two distinct reactors; where the processes are occurring singly or side by side or in parallel [18].

Such bio-process has been shown to be effective in a number of food systems for enhancing yields of beneficial compounds from foods without compromising their sensory and nutritional quality [19], [20], [21]. Unlike the conventional heating systems where heating of the food is done by conduction, convection or radiation, Microwave technology allows the direct interaction of microwave energy with the materials thus converting to heat energy [20]. Many theses discovered microwave useful in improving extraction operations [16], [22]. Besides, microwave technology is known for its use in green chemistry, as the technology can easily sterilize bacteria and enzymes when treating wastewater in the extraction business, making it effective in its operations [23]. UAE is another significant approach improving environmentally friendly or what we refer to as ‘green’ chemistry for the food process. The UAE working through the process of acoustic cavitation leading to cell wall disruption of plant cells so that the bioactive compounds could be released. Ultrasound waves with frequencies ranging from 20 to 100 MHz are mechanical waves which create a cycle of pressure and rarefaction that cause formation of cavitation bubbles and their subsequent collapse within liquid-based media. This process improves several food processing techniques such as, fermentation, emulsification, crystallization, reaction kinetics and extraction [24], [25]. Application of ultrasound in food analysis and process enhance the mass transfer rates, mixing efficiency, energy utilization, production rates and rejection of unwanted temperatures. Due to its versatility, it is well suited for the dairy application where it is employed in the laboratory systems for physicochemical properties, monitoring and non-destructive examination. Aside from non-thermal processing, there has been the application of high intensity ultrasound systems in the fermented industries, extraction, pasteurization, and homogenization for the dairy processing [26]. Some of the benefits include low equipment and running cost, faster extraction time and high extraction rate, possibility of using low temperature, thus promoting environmental friendly extraction processes by improving on extraction effectiveness and minimizing solvent and energy utilization [27], [28]. Therefore, US-MW extraction effectively valorizes proteins from dairy wastes due to their novel nature and thus presenting a sustainable alternative to conventional methods. Also, US-MW techniques help in prevention of off-tastes and odors with less cost and labor-lessness.

## 2 Fundamentals of ultrasonication and microwave extraction

### 2.1 Ultrasonication

Ultrasonication is a physical and non-thermal processing technique widely used to extract bioactive chemicals. This technology is valued for its low investment costs, ease of installation, low maintenance requirements, energy efficiency, and cost-effectiveness. In accordance with the Shen et al. [29], ultrasonication increases the yields of extraction by declining consumption of solvents, time of processing, and enhancing the solvent efficiencies. Ultrasound (US) is categorized in high (i.e., 20–100 kHz) and low (20 kHz to 1 GHz) intensities. The technique utilized these sound frequencies to generate acoustic cavitation within a aqueous medium resulting in disruption of cellular structures [30]. The steps involved in Ultrasonication-assisted extraction are; cellular break down to increase hydration and decrease size, causing erosion and pitting on cellular surfaces, enhancing solvent penetration efficiencies, improving swelling and hydration, molecular detachment from surface of samples, and physical bonds disruption and breakdown into the smaller ones. Also, promoting the swelling and hydration by dissolving cells [31].

Ultrasound methodology has been used in several industries for different applications such as inactivation of microorganisms and enzymes, filtration, dehydration, degassing, crystallization, sterilization, extraction, homogenization, and oxidation [24]. In recent few years, ultrasound has extensively explored for its efficiency in proteins extraction from the plants, animals, and agro-industrial wastes to reduce time of extraction, consumption of energy and solvent uses [29]. Further, studies has elucidated the ultrasonication to alter microstructural, functional, and physicochemical attributes of proteins extracted by US. Cavitation impact of ultrasonication produces significant turbulent flow and shear force which can increase interaction between air and water and affects physical interactions such as hydrogen bonding and hydrophobic. The modifications can increase functional attributes of proteins like emulsifying ability, solubility, and digestibility [32].

### 2.2 Mechanism of ultrasonication: Cavitation and its role in breaking down cell walls

Cavitation the key process driving ultrasonically assisted extraction, was initially identified as causing inefficiencies in the propulsion of the high-speed torpedo boat HMS Daring [29]. Early research focused on hydrodynamic cavitation, which is produced by propellers passing through water. However, power ultrasonication can generate a different type of cavitation. Like other sound waves, ultrasonication waves travel through a medium by creating rarefaction and compression waves that move molecules [33]. Cavitation bubbles can form when ultrasonic waves of sufficient strength are applied. This happens because the rarefaction phase of the sound wave can overpower the forces of attraction between liquid molecules. During the bubble’s expansion, a small amount of gas or vapor from the surrounding medium enters, but it is not fully expelled during compression. This process known as rectified diffusion, is what allows these bubbles to expand. When these bubbles ultimately burst during subsequent compression cycles, a significant amount of energy is released. Cavitation bubbles in a homogeneous liquid are typically symmetric [34]. However, when these bubbles collapse in the bulk liquid, they generate localized hot patches with extreme conditions, reaching pressures of approximately 200 atm and temperatures of 5000 K [35].

Nevertheless, when these bubbles burst near a solid surface, and the situation changes. This phenomenon was observed and studied many years ago [36]. The cavitation does not create vacuum and ultrasonic surface requires rigorous cleaning due to asymmetric collapse of bubbles of cavitation and generates high velocity jets (400 km/h) and removes pollutants, debris, particles, dirt and biofilms [37]. This phenomenon is a key reason for the effectiveness of ultrasonic surface cleaning. When cavitation bubbles collapse asymmetrically near a surface, they generate high-velocity jets capable of efficiently dislodging particles and contaminants, often reaching speeds over 400 km/h. this mechanical force is powerful enough to remove dirt, debris, and even biofilms from various materials, resulting in a thoroughly cleaned surface. The visual representation of both symmetric and asymmetric bubbles collapse underscores the significant mechanical impact of these fast-moving jets as they strike clean surfaces [38]. Cavitation do not cause vacuum owing to diffusion which allows bubbles to generate by gathering dissolved gasses and solvents vapors and creates reactive hydroxyl radicals (OH^.^) and hydrogen atoms. Free radicals generates hydrogen peroxide (H_2_O_2_) and oxidizing agents [4].

Elevated levels of ultrasonication results in extract degradation. Inert gases like argon or nitrogen can be bubbled into mixture in ultrasonication assisted herbal extraction to decrease cavitation collapse energy. Further, using ethanol or water mixtures as extraction solvents can also reduce the production of highly oxidizing species from water breakdown. This reduction occurs because water and ethanol both enter the cavitation bubbles during their formation through rectified diffusion, with water being less susceptible to homolytic cleavage than ethanol [29]. In a heterogenous extraction mixture, the main cause of cavitation is the asymmetric collapse of bubbles. This leads to the formation of solvent jets that rapidly target the plant material. Ultrasonically aided extraction (UAE) is highly effective due to this process. It is crucial to understand this aspect when developing potential UAE extraction procedures [39].

### 2.3 Role of ultrasonication in the dairy industry

The creation of new dairy products is becoming more important due to increased consumer demand for meals that taste better, as less processed, and are healthier. Another type of food processing, known as sonication or ultrasonic processing can be used to improve the functional and technical qualities of milk and dairy products. The ultrasonication has been used in the dairy industry for various purposes, including food safety, yogurt and fermented beverages production, homogenization, and emulsification of milk. High-intensity ultrasonication has minimal side effects, although there may be mild color changes and minor protein oxidation. However, the use of ultrasonically processed milk may result in an undesirable final product. Further research is needed to better understand the impact of lipolysis, fatty acid oxidation, and proteolysis on the sensory qualities that primarily affect quality. It should be noted that the ultrasonication alone is not effective in removing bacteria from milk: however, when combined with heat, it can effectively eliminate bacteria [40]. Power ultrasonography has been found to positively impact the mixing of oil-in-water emulsions when studying its physicochemical effects in an oil bath. Since its discovery, ultrasonication has been widely used in commercial food processing for various purposes, including energy-efficient food material cutting, homogenization of products like milk and mayonnaise, extraction of flavonoids, degassing, cleaning, microbial deactivation, and enzyme inactivation [41]. Studies have shown that ultrasonication is equally, if not more, effective than conventional food processing methods across all technical scales [42].

Studies have shown the various ways in which the dairy sector has utilized ultrasonication technology such as nondestructive analysis of milk and its components, increasing milk processing efficiencies, ensuring safe dairy products and microbial reduction and waste reduction. US-assisted UHT packaging sealing have been successful in the industry. Ultrasonication technology in synergy offers several benefits like higher quality, energy & mass transfers, and efficient extractions. Ultrasonication technique is highly recognized for its wide dairy applications in the extraction, and processing due to its rigorous processing time and pathogens inactivation [24]. In dairy sector, the ultrasonication is used owing to its better power and frequency and safer approach [30]. Ultrasonication is viewed as an effective substitute for enhancing the quality of dairy food products by inactivating enzymes and microbes as well as improving the yield, texture, and color of dairy foods [43]. Ultrasonication has revealed a consistent improvement in dairy products’ nutrient retention, shelf life, and edible quality of bovine milk. ultrasonication at higher intensities is linked with the improved curd matrix, fats, droplet size and gel formation. Hussain et al. [44] has validated plausible findings for the use of high-intensity ultrasonication resulted in significant improvement of milk and milk beverages.

Earlier, milk proteins have been modified using ultrasonication technique for improving the physicochemical, yield and sensory attributes. Earlier studies have validated the use of ultrasonication for improving the yogurts cohesive and gel structures compared to raw milk to obtain a dense gels with fine microstructures with large elastic moduli [45]. Additrionally, thermosonication at 24 kHz for 100–150 W at 70 °C stimulated whey-casein linkages in milk and decreased serum separations in yogurt beverages and soft cheese. Earlier research have portrayed the cavitation effect to play a crucial role in modifying dietary components. Low frequency and high energy ultrasonication (20–100 kHz) have been found effective then using at 20–25 kHz for modifying the structural size and shape of dairy proteins. Furthermore, it has been suggested that by employing the ultrasonication under appropriate conditions, interactions among food components can be improved without altering the fundamental structure of the components. In comparison to high-pressure processing (HPP) and microwave (MW) treatments, the ultrasonication has additional activities that mainly affect the structure of native casein and whey proteins. These activities include the dissolution of previously denatured protein clumps in suspensions due to the shearing impact of high-power, low-frequency ultrasonication and the chemical destruction of proteins caused by radicals created by low-power high-frequency US. It has been claimed that aggregates in suspension collide with more force because of the shock waves and microjets generated by ultrasonic technology. It is believed that the ultrasonication disrupts weaker hydrophobic connections or Van der Waals forces that hold protein clumps together, resulting in homogenized protein solutions. Hydrophobic interactions keep protein clumps together in whey protein concentrates (WPC) made from cheese whey. Recent studies show that sonicating a reconstituted 5 % (w/w) WPX solution at a frequency of 20 kHz and power of 31 W for 60 min can reduce the average particle size from 200 to 125 nm. Similarly, it has been found that sonicating a suspension of reconstituted whey protein isolate at a frequency of 20 kHz and power of 34 W/cm^2^ for two minutes can lead to a 50 % reduction in aggregate size [46]. Further research has shown that solutions of sonicated milk protein concentrate, sodium caseinate, and denatured casein-whey protein mixture also experience comparable reduction in aggregate sizes when treated with ultrasonication at a frequency of 20 kHz and an electric power of 300 W or a power density of 34 W/cm^2^. Subsequent studies examined the effect of bath ultrasonication (40 kHz, 1 W/cm^2^) and probe ultrasonication (20 kHz, 43–48 W/cm^2^) on reducing aggregates in WPC. Probe based ultrasonication results in higher cavitation zone and prevents energy losses and give best results [46]. Also, the ultrasonication at higher intensities of radiations can modify dairy proteins by disrupting casein micelles and by decreasing the size of micelles. Pugliese et al. [47] reported the skim milk particles size reduction by ultrasonication at 22.5 kHz and 50 W for 10 min. Another study elucidated ultrasonication to uphold better potential to dissociate casein micelles [40]. Furthermore, milk was sonicated at 20 kHz and 286 kJ/kg power for 15 min at a high pH, followed by neutralization. Casein micelles were found to be expanded at higher pH owing to electrostatic repulsions, which enables higher susceptibility towards mechanical forces [48]. Ultrasonication of caseins still needs further exploration, earlier studies have reported the whey proteins to undergo several structural modifications on US. It has been reported, sonic shear at 20 kHz and 31 W resulted in unfolding of whey proteins. However, at over 10 min of ultrasonication the results showed the whey proteins to form aggregates owing to hydrophobic interactions [49].

### 2.4 Mw-assisted extraction

The extraction process related to the use of MW has been highly approved for its effectiveness as well as being systematic to sustainable ‘green’ chemistry and extraction. In the food and chemical industries, it has been used for the pretreatment processes and identified to have the ability to extract different bioactive compounds such as proteins, pectin and phytochemicals [50]. Valverde et al. [51] pointed that this method involves the use of electromagnetic waves that moves in the range of 200 MHZ to 300 GHz. Like the light and infrared waves, MWs also has the properties of absorption, transmission and reflection. Jointly, the electrical power is used and a MW power source or generator is used to offer MWs in MW-assisted extraction.

### 2.5 Mechanism: Dielectric heating, polarization and disruption of cells

It consequently implies that the dipole rotation and the ionic conduction collaboratively disrupt the hydrogen bonds in cells of the plant. This is the basis for the efficiency of MW technology of biomolecules such as proteins extraction [52]. This disruption increases the hydrophilic nature of cell membrane, thus the solvent can penetrate through the cellular structure and liberate other chemicals that are inside the cell. The mechanism of its operation apparently involves passage of electrical energy at the molecular level by the electromagnetic field inside the chamber of the device. the movement of polar particles in the electric field and the acceleration of ions (ionic conductivity) are characterized by a high frequency of interaction with water molecules, which increases the frictional forces leading to the release of thermal energy [53]. Increasing the penetration of the solvent into the matrix and the subsequent disruption of cell walls and the subsequent liberation of intracellular products, the rotation of dipoles and shift of ions by MWs superimpose the extraction of specific molecules [54]. In general, it is obvious that there are certain advantages of MW-assisted extraction in comparison with conventional methods. Such are faster heating, short processing time, high reproductibility, low solvent consumption, easy handling, efficient heating, effective heat transfer, low thermal gradients, localized heating and higher product recovery [55]. Some of the factors affecting this extraction process include cell wall structure of plant and quality solvents used, the solid solvent ratio, power, time taken and the temperature used in passing the microwaves [56]. Xu et al. [57] have experimentally shown that heat distribution within a heterogenous food matrix can be not consistent, which results in random breakdown of proteins and subsequent decline in conductivity. The study done by other researchers has also demonstrated that proteins extracted by MW assistance have a higher biological functioning as compared to conventional solvent based methods.

### 2.6 Application of MW in dairy industry

In dairy processing raw milk is normally subjected to certain processes including pasteurization freezers and driers. These processes yield products such as butter, milk, ice cream, yogurt, dried milk and cheese among others. But the waste that accrue from these procedures has a lot of consequences to the environment. This effluent comprises dissolved carbohydrates, lipids, proteins, minerals and some other organic matters of milk [58]. Proteins are essential ingredients of dairy foods which contributes to improve the edible qualities. However, these components are susceptible to degradation and microbial attack, necessitating techniques that minimize the loss of nutritional and quality attributes. Milk, a complex multi-nutrient food, is particularly vulnerable due to its high nutritional value and moisture content, making it prone to spoilage by pathogenic microbes. The main nutritional components of milk include lactose, fat, proteins (casein and whey), minerals, and vitamins [40]. Traditional heat treatments are commonly used to kill harmful bacteria in milk, which is often contaminated during the milking process. However, commercial heat treatment can degrade the quality and nutritional content of milk, leading to the loss of polypeptides, vitamins, and other desirable attributes like flavor and color. High temperatures used in thermal processing, while effective in inactivating bacteria, also break down volatile and heat-sensitive components, resulting in significant sensory and nutritional losses in dairy products [59].

MW is a considered as a viable heating method as it provides heats by affecting the polar water molecules and charged ions in foods and results in higher temperatures and moisture penetration. This method is preferred in the dairy processing for pasteurizing without much heat addition that increases the heat load and reduces the quality of the milk proteins and enzymes [60]. MW systems are well recognized for their capability in retaining those heat-sensitive nutrients including ascorbic acid, B vitamins, and antioxidants besides reducing the processing time and costs. Compared with the conventional techniques microwave extraction has been proven as efficient method while helpful in reducing pathogenic microbes, and enhance treatment efficiencies with improved food qualities. For instance, MW causes the unfolding and denaturation of casein and whey proteins more rapidly than conventional heating, leading to harder and more rigid acidic gels due to increased cross-linking [40]. MW categorized as non-ionizing radiation, can induce both thermal and non-thermal changes in milk by altering the molecular structure of proteins through friction and molecular rearrangement. These effects can modify the quaternary and tertiary structure of proteins, impacting their functional properties the exact influence on protein and enzyme function remains a topic of ongoing research [49].

## 3 Synergistic effects of combined ultrasonication and MW extraction

When used together, multiple extraction methods can effectively overcome the limitations associated with using each method individually, thanks to the process of synergism. This technique involves use of more methods consisted of variety of instruments for improving speed and results [29]. Significant requirements for obtaining the synergistic process include enhancing molecular interactions, maintaining homogenous mixing and heating, improving heat and mass transmission, and enhancing the effectiveness of traditional processes [61]. It is important to configure the processes, sources of energy, molecular structure and process control [29]. But, obtaining process precision and optimization of processes is difficult [61].

### 3.1 Synergistic effects of microwaves and ultrasonication in dairy industry

Ultrasonication and microwave synergistic use is recognized in dairy sector for efficient use in pasteurization offering nominal to zero waste and improve physicochemical quality of milk proteins and milk enzymes [40], [62]. Unlike conventional thermal methods, microwave dielectric heating provides enhanced volumetric heating, leading to improved product quality and greater thermal efficiency. Microwave heating systems have demonstrated their ability to produce high-quality milk-based products with extended shelf lives, positioning them as viable alternatives to traditional heat treatments. The advantages of microwave technology in the dairy sector include rapid heating, high thermal performance (at least 80 % efficiency), volumetric heat generation, and a reduction in processing time by approximately 75 % compared to conventional methods. This rapid heating capability minimizes adverse effects on sensory attributes and nutritional quality [40], [63].

While, ultrasonication is a non-destructive method that offers numerous benefits such as accelerated processing, improved efficiency and elimination of certain steps while maintaining the quality of products including nutritional value, texture and organoleptic characteristics with elongated preservation [40], [42]. As an emerging technology, ultrasonication is being effectively applied across various food processing operations, including separation, freezing, extraction, filtration, emulsification, drying and sterilization. Research indicates that ultrasound technology enhances the efficiency of existing processes and improves the quality of food products [24], [40]. When combined with other technologies, ultrasonication provides advantages such as accelerated energy transfer, improved food quality retention, enhanced mass transfer, reduced concentration and thermal gradients, and increased production efficiency, effective extraction and a cost-effective alternative to traditional thermal methods [40].

### 3.2 The role of combined technologies in improving yield and purity

The novel hybrid technology, known as ultrasonic-microwave synergistic extraction (UMSE), combines the strengths of MW and ultrasonication methods. By harnessing the enhanced mass transfer from ultrasonic cavitation and the high-energy impact of MW, UMSE overcomes the limitations of traditional US and MW extraction techniques [64]. Also, UMSE enhances the solvent dissolution and dismantling of components, and at the same time heats up the extraction matrix at a fast and constant rate. Various problems associated with uneven heating in MAE and insufficient thermal impact in UAE are solved through the synergistic use of UMSE. ultrasonication assists in phase advance from the solvent into the sample matrix, resulting in destruction of cells with enhancement of surface area, mass transfer and enhances rate of dissolution of soluble components [29].

### 3.3 Review of relevant studies

Whey protein-rich fractions were recently investigated to examine the effects of ultrasonication at 20 kHz, 75. 6 J/mL on the secondary structure of whey proteins. The results revealed the β-lactoglobulin (β-LG) dimers to be separated and random coil conformations within the monomers were higher due to ultrasonication. Also, ultrasonication was identified to decrease the extents of free sulfhydryl groups in isolated solutions of bovine serum albumin (BSA). The study also observed an increase in thiol contents of WPC which could be as a result of the unfolding of proteins [46]. Transformations in functional attributes of dairy wastes on ultrasonication are attributed to protein conformational changes and homogenization. Protein aggregations are reduced due to the reduction in turbidity and viscosity [61]. Solubility and viscosity during ultrasonication have been reported to be altered due to protein denaturation and aggregations. The solubility and flow ability of whey protein isolate solutions were shown to be reduced by ultrasonication (30 kHz, 73–78 W/cm^2^, 15 min) [65]. Free caseins re-aggregate with casein micelles under increasing sonic cavitation shear pressures. It has been discovered that the turbulent circumstances created by ultrasonic therapy improve particle mobility and encourage the development of aggregates [49]. Research conducted on the structural and thermal characteristics of proteins in reconstituted WPC solutions showed that extended ultrasonication, which leads to protein aggregation, increases the enthalpy of denaturation, whereas ultrasonication for as little as five minutes decreases it. Despite causing minimal changes to the protein’s hydrophobicity and secondary structure, ultrasonic processing preserved the functional properties of dairy products based on whey protein. The effect of low-intensity ultrasonication has been studied in various areas including microbial development, enzymatic reactions, fermentation control, gelling processes, and the characteristics of rennet coagulation in milk. In cheese manufacturing, the ultrasonication has been shown to reduce the time it takes for curd to set and increase curd stiffness [40].

### 3.4 Comparison with traditional extraction methods

MW heating has been shown to require shorter treatment times than conventional thermal pasteurization to achieve the same level of microbial deactivation. This is because cellular proteins break down faster under MW heating. As a result, MW-assisted pasteurization is effective in reducing the extent of undesirable biochemical changes that typically occur in milk during thermal pasteurization [49]. For instance, Murtaza et al. [40] found that MW-pasteurized milk has satisfactory quality, with reduced enzyme activity and less deterioration of milk components. D'Incecco et al. [66] also reported improved sensory attributes in MW pasteurized milk, such as better order, caramelization, astringency, and reduced fatty flavors compared to milk treated with UHT.

According to research, using MW for dielectric heating improves volumetric heating compared to conventional thermal techniques. This results in higher-quality goods and increased thermal efficiency. Studies have shown that milk-based products heated in a MW system yield superior products with longer shelf lives, making them a viable alternative to traditional heat treatments [67]. However, it is important to note that MW heating can lead to nonuniform temperature distribution, especially with semi-solids and solids, despite this limitation, MW heating is considered suitable for various liquid meals, especially in continuous fluid systems. MWs are highly efficient at rapidly heating and transforming food into heat, which is beneficial for the dairy sector. They heat food at least 80 % more efficiently and process it in up to 40 % less time compared to previous methods [68]. Additionally, MWs help to maintain the quality of processed meals better. These quick heating rates make MW particularly useful for heating fluids that have high viscosity, are multiphase, or are thermally sensitive. MW reduces the negative effects of heat on sensory and nutritional qualities. Furthermore, MW heating enables the safe handling of pasteurized items after they have been packed [69].

Compared to High-pressure processing, which primarily affects the native conformation of casein and whey proteins, ultrasonication offers two additional functions: (a) the disintegration of already denatured protein aggregates in suspensions due to the shear forces generated by high-power, low-frequency ultrasonication, and (b) the chemical degradation of proteins caused by radicals formed during low-power, high-frequency US. The ultrasonication produces shock waves and microinjectings which results in increased collisions among protein aggregates in the suspensions, which results in fragmentations. This is even more pronounced, when the aggregates are stabilized due to poor interactions such as hydrophobic interactions or van der Waals forces. For instance, the use of ultrasonication to lower the size of aggregates in whey protein concentrates (WPC) is highly effective [49]. Jambrak et al. [70] sonicated dairy waste at 20 kHz and 31 W, and the results revealed significant reduction in the particle size of reconstituted WPC solutions from 200-125 nm at 60 min. Murtaza et al. [40] revealed the ultrasonication at 20 kHz and 34 W/cm2 for 2 min to reveal appreciable size reduction by 50 % in WPI, sodium caseinate and denatured casein whey proteins. Asadi et al. [71] probed ultrasonication to have lower aggregates in WPC than bath ultrasonication because the former generated a longer and more intense cavitation zones. The degree of size reduction possibly depends on energy interactions during cavitation per unit area. Table 1 compares the conventional extraction methods with US-MW extraction, highlighting the efficiency, environmental impact, and cost-effectiveness of the latter.

**Table 1:** Comparative assessment of conventional and US-MW extraction.

**Criteria**	**Conventional Extraction**	**US-MW Extraction**
**Separation efficiency**	Opposed by surface-active compounds, leading to emulsion development during extraction	The phase-forming constituents successfully maintain a low interfacial layer between the phases, resisting emulsion formation
**Processing time and speed**	The conventional extractions are relatively slower processes and consume more time for recovering valuables from the wastes	The novel techniques are faster methods which come with better extraction efficiencies
**Energy consumption**	Substantial energy inputs are required	Comparatively low energy input needed
**Feasibility**	Complex process	Simple and easy to operate
**Running cost**	Costly	Comparably less expensive
**Interfacial tension**	Higher interfacial tension (ranged 1–20 dyne/cm)	Lower interfacial tension ranging between 0.0001 And 0.1 dyne per centimeter
**Protein extraction rates**	Conventional methods, for example, salt and alkaline extraction, showcase less protein recovery attributed to low rate of extraction and high solvent consumption.	The novel extraction technologies including subcritical, ultrasonic and microwave have been shown to give higher protein extraction rates
**Solvent utilization**	Large volumes of solvents are used for better extraction outcomes	Better extraction capabilities using low volumes of solvents
**Environmental sustainability**	The large volumes of solvents used contain non-recyclable reagents which must be dumped into the environment, posing enhanced risks associated with environmental sustainability	The novel techniques use non-toxic phase forming constituents which are environment-friendly as compared to conventional solvents
**Selectivity**	Limited selectivity, especially for complex mixtures	Can improve selectivity by targeting specific compounds based on their physical and chemical properties
**Scale-up**	Can be challenging to scale up to industrial production due to equipment limitations and energy requirements	While scaling up can present challenges, advancements in engineering and technology have made it more feasible
**Safety**	Requires careful handling of chemicals and other solvents	Require careful control of parameters to avoid cavitation-induced erosion and safety hazards

### 3.5 Advantages of the combined approach

In comparison to the conventional techniques of extraction, ultrasonication and microwave extraction have recently emerged as novel techniques involving radiation and ultrasonic therapies accompanied with higher extraction rates and fast processing time. ultrasonication extraction is supposed to be better because of its efficiency of better cell disruption ability, higher mass transfer, good diffusivity, and capillary impacts. However, microwave extraction is also known for its remarkable extraction ability and time efficiency. However, these two techniques reveal quick extraction, better utilization of solvents and energy, and cost effectiveness [72]. It is claimed that the extraction process is accelerated and the extraction time is shortened due to the synergetic effect of amplifying heat transfer by MW and mass transfer by US. This method is also believed to be more ecologically beneficial as it uses green solvents and has a quicker reaction time [29]. Table 2 outlines the changes in milk protein properties induced by ultrasonication.

**Table 2:** Milk proteins properties as affected by ultrasonication.

**Properties**	**System**	**Treatment**	**Impact**	**Reference**
Emulsification	Reconstituted milk protein concentrates	15 W, 20 kHz, 5 min	The emulsion stability index initially increased during the first minute of treatment but then decreased between 2 and 5 min	[13]
	Reconstituted whey protein isolate	75 W cm^−2^, 20 kHz, 30 min	The first fifteen minutes of treatment saw an increase in foam stability, which decreased between the fifteen- and thirty-minute mark	[147]
Viscosity	Sodium caseinate reconstituted	34 W cm^−2^, 20 kHz, 2 min	Viscosity was reduced	[148]
	Whey protein isolate reconstituted	31 W cm^2^, 20 kHz, 20 min	Surface hydrophobicity was increased	[82]
Protein aggregates size	Skim milk	50 W, 23 kHz, 10 min 286 kJ kg^−1^ power, 20 kHz, 15 min	The size of the casein micelle was reduced After being sonicated at pH 8 and neutralized to pH 6.7, the size of the micelles decreased.	[149]
Free sulfhydryl groups	Whey protein concentrates reconstituted	4 W, 20 kHz, 20 min	There were no changes observed in free sulfhydryl groups	[49]
Turbidity	Whey protein concentrate reconstituted	50 W, 20 kHz, 1 h	Turbidity was reduced	[65]
Surface hydrophobicity	Whey protein concentrate reconstituted	31 W, 20 kHz, 60 min	Raise to five minutes, then reduce because of aggregation from five to sixty minutes	[48]
Solubility	Whey protein isolate reconstituted	31 W cm^−2^ and 69 W cm^−2^, 20 kHz, 20 min	Solubility was increased	[65]

## 4 Applications of extracted proteins

### 4.1 Nutritional and functional properties

Milk and dairy products are essential nutrient foods from which man derives some of his nutritional needs as has been provided in several code of nutrition in different parts of the world. They provide a good package of nutrient matters which include important micro and macro elements such as calcium, magnesium, selenium, riboflavin, zinc, vitamin B_12_ and pantothenic acid [61], [63]. Also, milk and its products are rich in whey proteins, vitamins, types of casein, organic acids, bioactive peptides, easily absorbable calcium, antioxidants, lactoperoxidase, glycomacropeptides, conjugated linoleic acid, oligosaccharides, lactoferrin and sphingolipids these bioactive compounds are helpful to prevent cardiovascular diseases, gastrointestinal disorders and cancers [63]. As indicated in Table 3, certain dairy by-products, such as whey and buttermilk, contain high levels of protein suitable for extraction and functional use. These proteins found in milk consumption for purposes of glucose control, muscle build up, weight regulation and satiety control. The use of milk proteins in manufactured foods has therefore been identified as the most actively growing segment in the food processing industry. In addition, the global market for milk proteins has registered a significant increment in the last decade and has a brighter future for the future population growth world [73], [74].

**Table 3:** **Protein content of some dairy waste and by-products** (© British Nutrition Foundation 2018).

**Dairy waste and by-products**	**Protein Content (g/100 g)**
Whole milk	3.3
Semi-skimmed milk	3.4
Skimmed milk	3.4
Cheddar cheese	25.4
Cottage cheese	12.6
Whole milk yogurt	5.7
Low fat yogurt (plain)	4.8

The methods of ultrasonication and microwave assisted extraction gives new possibilities for enhancing the functional and nutraceutical properties of milk waste proteins as well as for the production for bioactive peptides. This approach employs the use of relatively low frequency of ultrasonic waves of between 20 and 100 kHz at high power levels [75]. Recent development of extraction method puts into consideration the integration of different techniques that can offer safer, non-toxic and eco-friendly extraction. Compliance of different extraction methods i.e., microwave-assisted extraction (MAE) and Ultrasonic-assisted extraction (UAE) may lead to higher protein extraction, better quality and shorter time [76]. In the dairy field, ultrasound and microwave treatment have risen fame as affordable techniques for making the microbial quality of dairy products superior than thermal methods [75], [77]. These innovative approaches also offer possibilities to alter the structural and functional properties of milk proteins, thus making the bioactive peptides to be released [75]. Also, it was shown that various non-thermal methods such as ultrasonication positively affect the digestibility of milk proteins. It has been postulated that ultrasonic treatment of fresh bovine milk (10–50 W/l, 10–60 min, 30 °C) promotes protein hydrolysis during in vitro gastrointestinal digestion more effectively as compared to untreated milk [78], [79]. Furthermore, [80] indicated that whey protein concentrates underwent positive changes consisting of enhancements in the in vitro gastric digestibility of the whey protein concentrates following 15 min of ultrasonication (24 kHz, 400 W). Camel milk whey and casein protein solutions were subjected to ultrasonication for a duration of 45 min at ambient temperature under 30 kHz frequency and 400 W power of a bench top sonicator. The antioxidant activity of whey and casein proteins after ultrasonication appeared to be enhanced and were rated at 27 % and above while the non-sonicated controls had antioxidant activity of 0.98 and 0.84 %, respectively. Other studies also concluded that there was an enhanced total antioxidant activity of skim milk post ultrasonication improvement that was complimented by the antioxidant properties of casein fraction [75]. In the same manner, it has been found that ultrasonication increases the DPPH free radical scavenging capacity of whey protein isolate [75], [81]. Ultrasonication was found to increase the functional and biological properties of camel milk casein and whey proteins in an essential manner [75]. Increase in the ultrasound exposure time also significantly improved (*p* < 0.05) both the DPPH and the ABTS radical scavenging capacities of whey protein isolate as compared with the non-sonicated sample and this corroborates the effectiveness of ultrasonication in generating whey protein fractions with higher antioxidant potencies [82].

Non-stop microwave treated milk described higher organoleptic properties than the indirectly described UHT treated milk but they did not show much variations between their microbial, and biochemical properties as described by [20]. Additionally, the [82] reported the enhancement in surface hydrophobicity, foaming and emulsifying property of the sonicated proteins. As will be discussed in detail all sonicated samples displayed a decrease of the particle size compared to the untreated whey protein isolate. The particle size was reduced most significantly after ultrasound treatment for 40 min with the size reaching the peak of about 157 nm. Similarly, [75] have also showed that the ultrasonication at the frequency of 30 kHz causes an alteration in the particle size distribution of whey proteins and caseins from camel milk by increasing the proportion of moderate size particles while reducing the z-average diameter by half [75], [82]. These observations indicate that the mechanical forces occurring under cavitation during the ultrasound processing, such as shear stress and turbulence, affect the breakdown of large WPI aggregates into smaller protein aggregates [82].

### 4.2 Potential uses of extracted proteins in food products

**Fig. 1:**
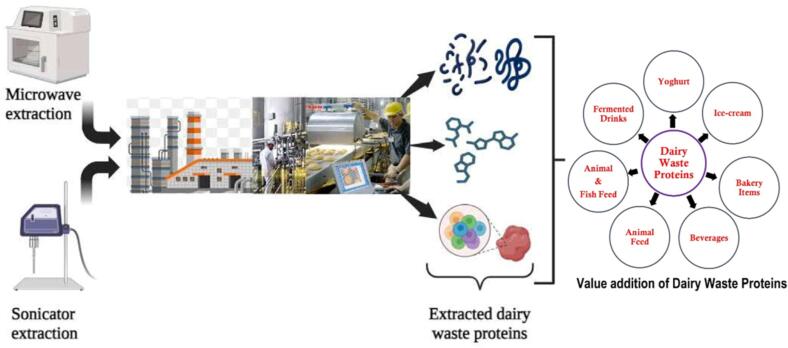
Industrial application of extracted proteins from dairy waste.

Today, studies, along with improvement of extraction methods and the access to sophisticated analytical tools, has led to a new focus on precisely extracting protein products from the whey. The product obtained after fractionation of whey proteins are known as WPs; which possess tremendous functional, therapeutic and nutritional properties making them highly desirable in the food industry. Moreover, WPs exhibit vital functional properties: such properties as solubility, foaming, emulsifying and gelling and water binding properties that greatly affect the structural and rheological characteristics of the final products. From the point of view of the highly advantageous properties of these extracted proteins, they turn out to be indispensable ingredients that takes a strategic role in the launching of new foods (El‐Aidie & Khalifa, 2024). Whey and its protein concentrates are popular in food production because of the nutritional and functional benefits offered by these ingredients. Being concentrated whey products, WPI and WPC contain low calorie, low fat, low sodium, good source of amino acids and protein. In addition, they are safe and free from hazardous compounds and therefore they can be used in foods in a variety of ways. Also, whey proteins are highly accessible, and its cost is reasonably cheaper [83], The various industrial applications of extracted dairy proteins are depicted in Fig. 1.

#### 4.2.1 Meat and fish products

The WPC and WPI are very central in the firming of meat products especially the comminuted products like sausage, mortadella and luncheon meat. These WPs affect the stability, biting and chewing the characteristics of meat products since their viscosity and water-binding capabilities reduces mass loss during thermal processing and storage besides improving the juiciness and slicing ability in cold meat products. Due to their greater solubility in the pH scale of 2–10, they are particularly suitable for use in injectable products and the tendency to from stable emulsions is essential in food product processing (El‐Aidie & Khalifa, 2024); [84]. WPC-80 has been reported to have the potential of being used as an effective replacement of powdered egg white in minced fish products and also popular Japanese foods such as surimi products (El‐Aidie & Khalifa, 2024); [85]. In addition, WPC has also reported as having excellent gelling properties that enhances fish protein gel and also for water binding and also give a whiter and glistening look to the product. Thus, whey proteins should be incorporated into edible sausage casings as it improves gelation additionally boost the taste of the meat product. WPs are also used to improve the batter holdings to meat, poultry and fish portions in such semi-finished products (El‐Aidie & Khalifa, 2024). In a fascinating study that [85] WPC incorporation into smoked chicken sausages prepared from broiler waste hens led to the improved frying loss. Another research showed that deep‐fried meatless patties containing textured WPC‐80 and mushrooms were accepted by the customers as per their expectations (El‐Aidie & Khalifa, 2024). Zandona et al. [86] studied the earlier research whereby the researchers blended WPC and WPI and develop high amount of mortadella.

#### 4.2.2 Fat replacers in lowered fat products

Lipids are used in food preparation for purpose of taste enhancement and improvement of textural profile, but because the high intake of animal fat might be detrimental to health, various organizations have recommended that fat be used in moderation. For this reason, replacement of high fat foods with low fat foods is encouraged. Therefore, WPC has been considered as an efficient fat replacer and it has added advantage of low caloric value when compared to animal fat (El‐Aidie & Khalifa, 2024). In a previous study [87], [88] found that WPC behaves like fats, since it can swell water and mimic fats in the mouth and therefore it is a fat replacer. In today’s food industries, egg yolk, soy protein and hydrocolloids are substituted with WPC-80 and PC-34 fat replacers. Since WPC have functional characteristics akin to fat products, as such, WPCs are being incorporated in many food products i.e., sauces, mayonnaise, soups, salad dressing, meat, ice cream and yogurt. WPC-34 and carrageenan can successfully be used in the blending of high quality low-fat sausages and this was found out by a research. Nevertheless, when incorporating WPCs as fat replacer, attention should be paid to the content of other additional spices and flavoring agents that are added to the product in order to enhance its taste. Studied showed that WPC used as fat replacer in nonfat goat milk yogurt, and the incorporation of fish oil with WPC results in yogurt with textural and sensory properties similar to those of full fat milk yogurt (El‐Aidie & Khalifa, 2024).

#### 4.2.3 Bakery items

Due to the functional and nutritive values of whey, this product has found its way into the bread, confectionery and biscuit industries. Whey contains high amounts of essential amino acids and as such it plays an important role of being a functional food supplement that provides a significant source of protein. It used mainly to replace flour or fat at baking commodities including breads, biscuits, cakes, cookies and many other kinds of baked foods and it give also creamier taste and improve texture and appearance. A number of research works have suggested that the addition of milk proteins to bread can have a beneficial effect on crumb characteristics, specific loaf volume, and taste and keeping quality; therefore, it can be considered as having potential use in bakery products. WPs have a significant function of improving the nutritional quality of high-protein biscuits and improving their sensory characteristics because of which the biscuits are preferred over other biscuits (El‐Aidie & Khalifa, 2024). Such characteristics make them a good fit for bakery products as supported with regards to numerous literature [89]. According to [85], table salt should be replaced with whey derived minerals in bread with a maximum inclusion level of 3 %. The above approach delivered a healthier bread without having to sacrifice taste or quality. In the earlier research by [90], the authors have proved that replacement of eggs with WPC in blend with gel emulsifiers in its application to prepare the eggless spongy cake has an improvement effect. From this study an ingredient blend of 6 % WPC and 15 % emulsifier gel was found to produce acceptable taste and texture of the cake. It has been also found that WPC can be used in part replacement of eggs and fat in cookie formulations such as chocolate chip or spice cookies. Also, it is used in the confectionary industry where it replaces sucrose because of its abilities to improve the crumb texture, color and taste of products (El‐Aidie & Khalifa, 2024). In bakery it has been used for enhancing crust browning, quality of products and shelf life of products according to the studies conducted by [91]. In their review of the WPCs derivatives, their potential application [92], demonstrated that aerated confectionery and chocolate where WPCs could be used due to its foaming characteristics in the food industry. Wu et al., [93] made an assessment on the effect of varying WPI concentration on texture properties of wheat-based crackers. When 20 % WPI was incorporated into the bread dough, the viscoelastic properties of the dough were significantly improved, and the acceptability of the product also increased; this indicates the possibility of using WPI for the improvement of wheat crackers.

#### 4.2.4 Ice cream

They noted that replacement of milk with WPCs in the production of ice cream results to a situation whereby the production cost is brought to the lowest level without necessarily compromising the quality of the product. Thus, by minimizing the usage of costly milk powder and egg yolk, the ice cream manufacturers are in a good position to achieve high economics of scale while not compromising on the quality of the manufactured ice cream (El‐Aidie & Khalifa, 2024). According to [94] an ideal ice cream contains a smooth creamy mouthfeel with a highly rich flavor which was realized by the use of 50 % each of WPC and skim milk powder (SMP) with lactose-hydrolyzed SMP. More studies have revealed that such a replacement of SMP with WPC at this extent can lead to improvement in the flavor profile of the ice cream, lesser meltdown and preferred texture [85], [95]. The following are recommended by [95], [96] ways of including WPCs in ice cream blends; WP (2–3 %), WPC-34 (1.5–3 %), WPI (0.5–1 %), or WPC-60-WPC-80 (0.5–2 %).

#### 4.2.5 Yogurt

WPCs are widely used in the production of yogurt owing to their unique functional attributes: WPC-34, generally incorporated at a concentration of 0.7 %–2.0 % and WPC-80 at 0.5–0.8 % in mixed yogurt. Nonetheless, the application of these additives is clear while the adverse reactions is the potential negative impacts of these additives which is that; they negatively affect some of the quality characteristics in the event they are used in excess. Further, the researcher noticed that the use of WPCs instead of SMP in yogurt manufacture offers an improved gel strength of the solid yogurt; increased viscosity of the mixed yogurt, hence the decrease of syneresis of both yogurt types [97], [98], [99]. In addition, the authors noted that with the application of WPI in the production of yogurt, there are potential options for the decrease of lactose. Additionally, the author pointed out that demineralized WP was identified to have the propensity of enhancing the fabrication of yogurt fermentation process. However, the reduced levels of minerals may also have a negative impact on the gelation properties and the firmness of the gels, especially when using this type of protein; in such cases milk protein hydrolysates have to be added (El‐Aidie & Khalifa, 2024). Minj & Anand [92] further revealed in their current study that whey and WPCs could contain bioactive molecules that enable the growth of probiotic bacteria within the final product and in the human gastrointestinal system. These findings suggest that whey and WPCs may have potential to promote probiotic growth in human (El‐Aidie & Khalifa, 2024).

#### 4.2.6 Beverage and fermented drinks

Whey has been employed in preparation of both fermented and non-fermented beverages in the beverage industry. WPCs have a propitious solubility profile as enabling them to be compounded in several categories of beverages ranging from fruit juices, soft drinks, wine and aperitifs among others (El‐Aidie & Khalifa, 2024); [100], [101]. Whey based beverages (WBBs) are well known for their refreshing and light properties and therefore known to be real thirst quenching product [83]. They are also consumed for the nutrition benefits which are always associated with them and are less acidic than most fruit juices making them a healthier option than many other drinks. Compared to fruit juices, WBBs are considered to be healthier for the body and non-acidic thus suitable for human consumption. To overcome the issue of sedimentation during storage, WBBs are usually prepared from deproteinized whey [86]. These ingredients are mostly added to the cow milk to improve the organoleptic and nutritional characteristics of the products such as dahi, khoa, kefir among others (El‐Aidie & Khalifa, 2024). For an instance, [102] observed enhanced organoleptic qualities of Khoa, formulated from cow’s milk on incorporation of 5 % WPC solids. Several investigations have reported that WPCs WPI and whey permeate are rich sources of bioactive compounds that add value to the texture, flavor and quality of fermented milk beverages (El‐Aidie & Khalifa, 2024). Furthermore, these components are established to enhance the nutritional quality and the shelf-life of those products and at the same time, enhance the minimization of waste [101]. The incorporation of acid whey (AW) may be a viable and environment friendly approach for developing functional probiotic foods, especially for small scale dairy enterprises [103]. Further, [104] emphasized this concept of generating new and unique carbonated probiotic drink using whey and highlighted the opportunity to unlock new horizon in dairy products.

The use of whey in production of alcoholic beverages is a creativity of this by-product and has a lead to the production of whey beer, whey wine and whey champagne. These beverages normally have a small percentage alcohol content, that is, about 1.5 %. These are mainly made employs whey permeate which is characterized by low protein content and involves yeast fermentation using yeast such as *K. fragilis* or *S. lactis*. Due to the presence of minerals and lactose, whey is useful in beer production but problems may arise from the fats forming peace which interferes with the stability of beer foam as stated in the research conducted by (El‐Aidie & Khalifa, 2024). An example of the earliest alcoholic whey beverage is a clear and light-golden wine, the alcoholic of which is 11 % and which originated from deproteinized whey concentrates. The following wine highlights; It has no sugar added and is colorless [105]. Other products include soft whey beverages apart from the alcoholic products. These drinks are prepared from vegetable and fruit concentrates which is prepared from different fruits like citrus, mango, banana, pineapple, kinnow, strawberry, papaya, carrot, and moringa leaf as mentioned in the work of [83].

### 4.3 Potential for use in bioplastics, animal feed and other applications

Due to their flexibility, relatively low price tag and low density, there has been a dramatic increase in the utilization of polymers across different industries including packaging and construction from the middle of 20th Century. However, decades of loosed disposal control and the fact that many of these materials are characterized by low degradability, have contributed to terrible impact on environment, expressed by the formation of such phenomena, as, for instance, the Great Pacific garbage patch. Plastic production in the global market was estimated to be about 359 million tons in 2018; about 50 % of this is used to produce disposable products which take their way to the dump sites or fitness for burning or are spread over the seas and oceans [106]. Together, 75 % of the overall global plastic manufacture totaling 9.3 billion tons has been transformed into plastic waste while only about 21 % of has either been recycled or burned [106], [107]. To date, the price of bioplastics is still higher compared to conventional plastics, though, the price may decrease with a massive production of these polymers [106], [108].

Thus, using starch, cellulose and proteins recovered from food waste can be considered as a potential approach to create bioplastic, which will fit into the concept of the circular economy and improve the revalorization of food by-products [106]. Since the milk proteins are widely used in the food industries the wastewater that is produced when cleaning the dairy processing plants contains casein which can be further used in the production of bioplastics [9]. Bioplastics based on casein can also afford transparent, flexible and fully tasteless films while the water resistance of their films can be crosslinked. Likewise, bioploylastic resulting from whey, though in the same category as casein, can form insoluble films as a result of disulfide covalent bond formation. Bioplastics which contain whey and egg albumin proteins have been developed using compression molding, and their characteristics resemble those of zein bioplastics even though zein is more expensive that whey protein [106]. Food waste can therefore be regarded as a large protein source [109] that has the potential to use in the production of human edible foods and as animal feeds [110]. Reuse of expired products is in conformity with the ‘closed-loop system’ concept [11], [111]. A comparative analysis was conducted to assess the effectiveness of the utilisation of expired dairy products as an organic fertilizer field as compared to inorganic fertilizer to enhance the growth of wheat (*T. aestivum* sp. *vulgar*) [111]. Studies conducted in this regard showed that there was an increase of 22 % in total chlorophyll, chlorophyll-a and nitrogen, phospherous and potassium uptake when expired dairy powder was used as a replacement to inorganic fertilizer [110].

## 5 Environmental and economic impact

Dairy industrial activities have increased in production, this has brought about increased production of wastes, this calls for proper management. This is a global concern since the management of wastes involve lots of hassles related with industrial development, or urbanization hence entail capital intensive investment for treatment or disposal. Consequently, attention has shifted to regarding waste as a new resource with emphasis on the recovery of resources for improvement of social as well as environmental quality. Current research mainly focuses on recycling of dairy waste and wastewater for the recovery of proteins, bio-fertilizers, energy and various bio-products [112], [113].

### 5.1 Sustainability consideration

Infrared, ultrasound and microwave methods of food preservation have received a lot of attention due to their nutritive preservation efficiency. These methods also enable modification and communication with food products [40], [114]. Microwave heating, especially, is a new method of thermal processing which finds more and more application to increase the microbiological safety, shelf life and functionality of food products [40], [115]. Ultrasound, microwave and high-pressure treatments cause changes in the physicochemical and functional properties of food macromolecules depending on the technology and food matrix. These enhanced technologies relating to food components could either be positive owing to the changes that occur in the food during processing or negative, depending on the changes a food undergoes during processing [40]. The two methods, microwave and ultrasonication, are potential in dairy processing. Microwave can help to avoid the formation of off-flavors and odors typical for the traditional heat treatment methods and are much more efficient in decontamination of the dairy products by eliminating microbial hazard. Third, microwave processing has shown positive influence on gelation which is another advantage in the application of dairy products. While ultrasonication has the prospects to minimize the possibilities of adulteration and thus avoid damaging the quality of food products, it enhances the WHC of the raw dairy products, which directly influences their quality, and which is a time-saving technique in the dairy industry as opposed to other traditional methods [40].

Mahmoud et al., [116] also showed that milk when subjected to sonicating at 40 °C for 120 s reduced the residual activities of lactoperoxidase (LPO) and alkaline phosphatase (ALP). In another research, the antibacterial activity of microwaved bovine lactoferrin (bLF) was examined under different power settings of 440, 550 and 650 W and exposure time of 5, 10, and 15 s used by [117]. After heating at 450 and 550 W for 5 s bLF was found to possess antibacterial activity at 94 and 89 % of its immunoreactivity levels. In addition, [118] portrayed enzymatic hydrolysis integrated with microwave extraction at temperatures of 37, 50, 65 and 70 °C to hydrolyze whey proteins leading to the production of highly hydrolyzed bovine whey protein hydrolysates with low allergenic potential.

### 5.2 Dairy wastewater and its effects on the environment

The rising consumption of food and enhanced laws touching on environmental matters have posed a challenge to the treatment and disposal of wastes and by-products in the food chain as it leads to incurring more costs on its management. Dairy industry produces millions of tons of by-products every year and cheese whey is the major part of it. Cheese whey is the remaining fluid and for the one kilogram of cheese production about 9–10 L of whey are produced. If not appropriately managed, cheese whey can lead to serious environmental implications because cheese whey is characterised by high BOD as well as COD [119]. Whey is especially considered hazardous as it is composed of significant amount of organic compounds inclusive of nitrogen, lactose, nitrates, as well as casein. This type of wastewater is highly degradable since it has high BOD and COD which may reach up to 48 and 95 g per liter respectively; this cause high level of pollutants that may have negative impacts to the environment [120]. When discharged into the water bodies, it reduces dissolved oxygen, creating danger to water creatures, the environment and people. Furthermore, the high level of lactose poses an issue regarding cheese whey and second cheese whey since it cannot be valorized since they cannot be used as animal feed due to causing digestive problems in animals [119], [121]. Furthermore, dairy wastewater increases sewage fungus especially the sphaerotilus species because they feed on low molecular weight sugars such as lactose. This fungal growth results to formation of filamentous slimes in water systems [120].

### 5.3 Role in a circular economy and waste valorization

Possible measures for handling with dairy by-products not only consider methods for preventing environmental pollution, but there are also ways for finding economic value by adding these by-products to formulations of food products. In the past, valorization endeavors have focused on cheese whey which has contributed to the formulation of new functional food ingredients, nutraceuticals and dietary supplements as well as some products as fermented beverages, whey cheese and yogurts. The whey can be processed from cheese whey to produce whey powder, WPC, WPI, whey protein hydrolysates, delactosed whey, demineralized whey and whey permeates [119], [122]. Whey proteins in their discreet form are used in food processing since they possess physicochemical and nutritional characteristics that make them suitable as emulsifiers, gelling agents, water binders and foaming agents [122], [123].

β-Lactoglobulin (β-Lg) which is present in milk contributes to the essential amino acids and is used in the formulation of nutrition products for athletes and in the dietary market because of its higher nutritional value as compared to other proteins [119]. Furthermore, β-Lg is involved in emulsification, frothing and gelling, therefore, it is used in forming protein hydrolysates for several ingredients’ applications [119], [124]. Whey proteins such as α-Lactoglobulin (α-La) is added to infant foods to adjust the quantity of proteins present in foods to be as close as possible to the human milk [119]. Additionally, (α-La) has antihypertensive and antioxidant effects, acts as an anti-obesity agent and possesses anti-tumor capability in the form of BAMLET, which is human alpha-lactalbumin made lethal to tumor cells [121]. Both WPC and WPI are widely used in the various food processing sectors such as emulsification, gelling and foaming. The remaining whey which is produced as a by-product of this process is often used as a fodder for animals. These applications bring economic returns because of the myriad of valuable by-products that are derived from whey, which is gotten through method like microwave processing and ultrasonication [40].

## 6 Challenges and future perspective

### 6.1 Scale-up issues, industrial implementation and needs

If extraction techniques are to meet the increasing sustainability needs in industrial processes then it is crucial to adopt upscaling techniques and make proper selection towards the proper parameter settings [125], [126]. For which, scaling up extraction techniques remains challenging, and the scientific and analytical method has to be uncompromising [126], [127]. In addition, term related to scaling like up scaling, lab scale and pilot scale- include a broad range of extraction capacity and therefore, complicated interpretation of the upscale levels. Previous studies have looked at different aspects of scaling up extraction technologies intensified from laboratory to pilot or production scale in terms of instrumentation, batch or continuous processes, kinetics, cost and energy. Thus, it is necessary to pinpoint that, depending on several critical factors such as energy consumption, the extraction system design and setting parameters, the scale-up operations might be successful or unsuccessful. This is quite significant to point out that, while using energy also has certain methods of its consumption differs from the other depending upon the method that has been practiced to extract the energy [126], [128]. One more which is worth mentioning is type of instrumentation or design of extraction units. Today hundreds of companies worldwide supply a broad spectrum of technologies ranging from scientific laboratory models to pilot and industrial scale for modern extraction processes. High capacity extraction units like those used in pilot and industrial processes are most often development-oriented in order to achieve end-user specification. Thus, to enhance the credibility of the proposed upscale system design, a pilot scale assessment may be performed subsequent to the laboratory investigation in accordance with the related literature [126], [128].

The existence of a great amount of analytical and empirical data allows avoiding many costly and time-consuming experiments and pre-testing, which are usually costly and complicated. In addition, parameter settings or extraction criteria are important for achieving success or failure of the scale-up processes [126], [129]. Some of the numerous studies by knowledgeable persons have focused on diverse quality characteristics and parameter settings of numerous natural product resources for eventual extraction of a broad array of high-value compounds. Some of the factors that affect the scale-up operations include: a number of variables depending on the extraction techniques. For example, in microwave-assisted extraction of essential oils the dielectric properties, solvent type, energy density, microwave power level, sample particle size and microwave irradiation time are the main parameters that influence the electromegnetic mechanisms for scaling microwave technologies [126], [130]. The same applies in ultrasonic-assisted extraction where scale-up factors such as the ultrasonic density, form of the vessel, temperature in batch or flow mode, duration of ultrasound, the ratio of sample to solvent and ultrasonic power as highlighted by [131]. However, scaling-up has to satisfy two objectives, firstly, the economy involved in the extraction process, and secondly, quality of the extract obtained. Whereas laboratory scale extractions are normally performed at a smaller scale using smaller quantities of sample and solvent and over shorter periods of time, pilot or industrial-scale operations require a stringent technological and economic appraisal [126].

### 6.2 Potential drawbacks and limitations of technology

The MW heating process involved energy transfer through non-contact method where source of energy, that is, electromagnetic energy is converted to heat energy. This conversion occurs through two primary mechanisms: such as ionic conduction as well as dipole rotation. Namely, non-ionizing MW radiation creates heat through ions oscillations and dipoles rotation in molecular level. The merits of MW heating you, focus on increased energy penetration to the material being treated, minimized thermal nearby gradients and selective heating (El‐Aidie & Khalifa, 2024). Thirdly, MW heating in combination with moisture in biomaterials creates a condition of internal pressure in the cell walls and ruptures this pressure mechanically to enhance solvent penetration in tissues inside. This leads to improved extractive yields and selectively (El‐Aidie & Khalifa, 2024); [132]. However, MW heating has one serious flaw, namely, the problem of rather non-homogeneous heating where overheated and cooler zones can appear in semi-solid to solid materials. However, MW heating is useful in different liquid foods particularly whenever used in continual fluid systems [40], [63].

Ultrasonication has several problems that have hindered the food industry in adopting from animal derived products. Because food materials are of different types, the probe should be designed and manufactured with different geometries, operating conditions to afford the right results. Furthermore, ultrasonication may affect food quality and cause defects as well in a minor way for some of the food shelf life, color changes and vitamin, anthocyanin and other phenolic compounds degradation hence not suitable for all types of foods. Some issues are still being observed on the possible transfer of metal ions from the ultrasonication horn into the food matrix which cannot be easily resolved without innovations in the alloy and probe design [78], [133]. Similarly, certain algorithms like food decontamination require very long processing times in order to achieve the results which are not favorable to the food properties [78], [134]. Similarly, the employment of high powerful intensity or lengthy ultrasonication period for a matrix can result into greater oxidation or degradation of compounds present in the matrix that decreases efficiency and usability of ultrasonication [135].

### 6.3 Exploration of other synergistic combinations for biomolecule extraction

New developments have even revealed that MAE can be combined with other therapies including the ultrasound therapy. It is believed that this combination will increase the quality of extracted product and food preservation as well. However, poor setting of the parameters of the used ultrasound system may result to changes of the flavor and deterioration of the compounds in the extracted product. Therefore, subsequent theoretical and experimental studies are required to successfully implement the proposed combined approach in industry scale applications [136]. Garcia-Vaquero et al., [137] also established that when employing a similar scenario but using brown microalgae, UMAE was effective in yielding higher concentrations of phenolic compounds and antioxidants than employing either MAE or UAE in isolation.

It also important to note that there is a great potential to integrating UAE and MAE. They can also be placed in a single reactor or in two reactors which work in parallel or one after another [14]. Wizi et al., [138] employed a simultaneous UMAE extraction system, employing a direct in-situ configuration from sorghum husk. With this method not only were the extraction yields much higher but also the extracts that were produced were of even better dyeing characteristics. The same configuration was successfully used for isolation of prebiotic oligosaccharides from sweet potatoes [139] and formation of starch-polyphenol complexes from Lotus seed starch suspensions supplemented with green tea polyphenols [140]. In both applications, the combined irradiation technique gave better extraction yields than conventional methods [14].

### 6.4 Development of more sustainable and efficient extraction methods

Improved extraction efficiency and sustainability may be best realized through the emergence of new green protocols to extraction, which often incorporate methods. These objectives are achieved when UAE and MAE are used singularly or in combination depending on a given situation. They increase the overall efficiency of the extraction of active principles and oils, do lower the selectivity and increase stability of the final product and in case of extraction of natural flavors and tastes they preserve the volatility of it. UAE and MAE are scalable for pilot as well as industrial level and several reports have been published which compare its efficiency with other extraction methods. Also, these techniques enforce mechanical changes in plant tissues in terms of swelling and permeation of solvents into cell [141]. UAE has been demonstrated to be efficient for the recovery of different herbal and food components such as oils, proteins, polysaccharides and bioactive natural product. High-intensity ultrasound is incorporated in flow reactors dissolving or emulsifying primary and secondary metabolites into liquid, mostly water without altering the botanical constitution of the plant. This results in the generation of extracts that possesses highly elevated level of these metabolites. It is also ironed out that Flow UAE is particularly tailored for process intensification since it facilitates the generation of enormous multiple transducer reactors, which power at remarkably high densities [141].

Rotor-stator hydrodynamic cavitation reactors are efficient in the extraction process. These systems include the use of cylinders which adjust their positions to be in line with stator channels while they rotate at high speeds. The processed liquid is centrifuged radially within the cavitation chamber and exposed to pressure waves, for formation of cavitation. The formation of the cavitation bubble collapse releases shockwaves that enhance interaction force of the solid and the liquid phase [141]. A study conducted to evaluate the degradation effects of UAE on phenolic compounds, examining the stability of gallic acid under varying extraction parameters including: acidity, concentration, gas content, time and different solvents with respect to input power, frequency and temperature. UAE with other techniques such as supercritical CO_2_ extraction can be described as an effective technique of improving the rates and yields because of the solvation and particle reduction. Further, ultrasound may create agitation in SFE processes in situations where mechanical stirrers are inconvenient [141]. Research has recently described a new eco-friendly process called mano-thermosonication (MTS) for the recovery of proteins from *Arthrospira platensis* cyanobacteria. The developed method integrates heat, pressure, and ultrasound into the extraction process so that mass transfer and cell disruption could be effective, up to 2. 29-fold increase in protein recovery and high yields of the fundamental amino acids from the microalga were achieved [141].

This work demonstrates that MAE is efficient in sample preparation of both volatile and non-volatile compounds in plant matrices. It is of particular importance for the retrieval of phytochemicals including anthocyannins, flavonoids and saponins vital in the production of health products, cosmetics, and drugs. MAE selectively can be improved by the proper choice of solvents, and the recovery of dried matrices with better di-electrical properties [127]. Another technique, solvent-free microwave assisted hydro-diffusion combined with gravity (MGH) has also been used to isolate bioactive compounds from edible brown seaweeds; *U. pinnatifida* and *L. ochroleuca*. The extracted liquid has prospect of functional hydrogels that can be applied with antioxidant functionality. Although it must be mentioned that the efficiency of MGH is particularly notable with high-moisture plant matrices [141], [142].

### 6.5 Integration with other emerging technologies

Solid-state microwave disruption also known as SSMD is a sophisticated method of releasing bioactive compounds from plant matrices. Uniquely, SSMD does not require the use of any solvents for biomass pre-treatment unlike in the traditional MAE. Since this method interferes with the plant microstructure and makes intracellular moisture to absorb more microwave energy, this results to increased heating and expansion pressure due to moisture vaporization as explained by [141]. The researchers from [143] has established how SSMD aids in the extraction of antioxidants and antidiabetic compounds from the Java tea leaves. The use of recycled by products in functional ingredient production has a great prospect especially since the EU discards about 100 million tonnes of food waste per year. The trend is moving from the conventional waste disposal methods such as; incineration and composting to techniques that improve substance recovery from waste. Among these second generation of waste management strategies, Ultrasonic and Microwave assisted methods are unique. The ultrasonic probes, that have high power at working frequencies 16–30 kHz, can significantly reduce energy loss during extraction. Further, pulsed ultrasonic assisted extraction PUAE that has active and inactive cycles help to avoid heat generation hence useful in preserving heat sensitive biomolecules [141], [144] followed an ‘‘green’’ way for conversion of Canasta sativa bud by-products using water: ethanol: glycerol (50:20:30 v/v/v). Research has also been done on enhancing the eco-sustainability in olive oil making processes of recent years as noted by [145]. Microwave helps in the release of oil from vacuoles while also cutting on water usage hence minimizing on wastage [141]. Further, [146]observed that the use of high-power ultrasound before malaxation of the olive paste enhanced the oil recovery by 1 % and the extractability is enhanced by 5.74 % without adverse effects on the olive oil quality.

## 7 Conclusions

US-MW are validated as viable green extraction techniques which are employed for the extraction of proteins from dairy waste. These techniques have shown significant potential as green technologies which can valorize dairy food wastes proteins in value added food products. Practical application of US-MW techniques can help in prevention of the off taste and odor production when compared to the conventional thermal methods used for extraction. Also, alone further helps in mitigating adulteration issues, enhance water holding capacities and enzymatic activities which are considered as desirable in various dairy operations in the dairy industries. Likewise, microwave extraction has also shown its effectiveness in destroying pathogenic microorganisms and improve gelation properties of dairy proteins which are used in dairy industries for food value addition. US-MW techniques are considered as time efficient, however, both techniques require higher refining before use in dairy industries. Hence, this gap between academia and dairy sector is to be filled by further collaboration, research, practicability and public outreach and awareness. US-MW extraction demonstrates significant potential as a green technology for extracting essential proteins from dairy waste, hence, offering benefits over conventional methods. Further research is required to focus on assessing the cost-effectiveness, energy efficiency, and scalability of US-MW techniques in other food sectors than dairy.

## CRediT authorship contribution statement

**Muhammad Waseem:** Writing – review & editing, Writing – original draft, Software, Resources, Methodology, Data curation, Conceptualization. **Muhammad Rizwan Javed:** Writing – review & editing, Writing – original draft, Software, Resources, Methodology, Data curation, Conceptualization. **Khubaib Ali:** Writing – review & editing, Supervision, Software, Investigation, Formal analysis, Data curation. **Muhammad Saleem:** Writing – review & editing, Writing – original draft, Software, Resources, Methodology, Data curation, Conceptualization. **Muhammad Faisal Manzoor:** Writing – review & editing, Writing – original draft, Software, Resources, Methodology, Data curation, Conceptualization. **Muhammad Farhan:** Writing – review & editing, Writing – original draft, Software, Resources, Methodology, Data curation, Conceptualization. **Robert Mugabi:** Writing – review & editing, Software, Funding acquisition, Data curation, Conceptualization. **Aanchal Sharma:** Conceptualization, Formal analysis, Methodology, Resources, Writing – review & editing. **Gulzar Ahmad Nayik:** Writing – review & editing, Supervision, Software, Investigation, Formal analysis, Conceptualization.

## Declaration of competing interest

The authors declare that they have no known competing financial interests or personal relationships that could have appeared to influence the work reported in this paper.
